# *Haemophilus influenzae* type B vaccination and risk of childhood leukaemia in a vaccine trial in Finland

**DOI:** 10.1054/bjoc.2000.1382

**Published:** 2000-09-04

**Authors:** A Auvinen, T Hakulinen, F Groves

**Affiliations:** STUK – Radiation and Nuclear Safety Authority, P.O. Box 14, Helsinki, FIN-00881, Finland; School of Public Health, University of Tampere, Tampere, FIN-33014, Finland; Finnish Cancer Registry, Liisankatu 21 B, Helsinki, FIN-00170, Finland; Department of Public Health, University of Helsinki, P.O. Box 441, Helsinki, FIN-00014, Finland; Department of Biometry and Epidemiology, Medical University of South Carolina, P.O. Box 250551, Charleston, SC 29425, USA

**Keywords:** leukaemia, child, vaccinations, *Haemophilus influenzae* type b, *Haemophilus influenzae* type b polysaccharide vaccine diphtheria toxoid conjugate, clinical trials

## Abstract

Incidence of childhood leukaemia was studied among subjects of a trial comparing administration of several doses of a conjugate vaccine against *Haemophilus influenzae* type b (Hib) starting at an early age (three months) with a single dose given at the age of two years. Among 114 000 subjects, a total of 77 cases of childhood leukaemia were detected. The incidence of childhood leukaemia was lower in the early vaccination arm (relative risk 0.72, 95% confidence interval 0.46–1.13) than late vaccination arm, but the difference did not reach statistical significance. Our results suggest that early immunization against Hib may reduce the incidence of childhood leukaemia, but confirmatory studies are needed. © 2000 Cancer Research Campaign


British Journal of Cancer (2000) **83**(7), 956–958
© 2000 Cancer Research Campaign
doi: 10.1054/ bjoc.2000.1382, available online at http://www.idealibrary.com on IDEAL®

### *Haemophilus influenzae* type B vaccination and risk of childhood leukaemia in a vaccine trial in Finland

**A Auvinen[1,2], T Hakulinen[3,4] and F Groves[5]**

[1]STUK – Radiation and Nuclear Safety Authority, P.O. Box 14, FIN-00881 Helsinki, Finland; [2]School of Public Health, University of Tampere, FIN-33014 Tampere, Finland; [3]Finnish Cancer Registry, Liisankatu 21 B, FIN-00170 Helsinki, Finland; [4]University of Helsinki, Department of Public Health, P.O. Box 441, FIN-00014 Helsinki, Finland; [5]Department of Biometry and Epidemiology, Medical University of South Carolina, P.O. Box 250551, Charleston, SC 29425, USA

**Summary** Incidence of childhood leukaemia was studied among subjects of a trial comparing administration of several doses of a conjugate vaccine against *Haemophilus influenzae* type b (Hib) starting at an early age (three months) with a single dose given at the age of two years. Among 114 000 subjects, a total of 77 cases of childhood leukaemia were detected. The incidence of childhood leukaemia was lower in the early vaccination arm (relative risk 0.72, 95% confidence interval 0.46–1.13) than late vaccination arm, but the difference did not reach statistical significance. Our results suggest that early immunization against Hib may reduce the incidence of childhood leukaemia, but confirmatory studies are needed. © 2000 Cancer Research Campaign

**Keywords:** leukaemia; child; vaccinations; *Haemophilus influenzae* type b; *Haemophilus influenzae* type b polysaccharide vaccine diphtheria toxoid conjugate; clinical trials

A number of studies have previously shown a lower prevalence of non-BCG vaccinations among children with leukaemia than in controls (Kneale et al, 1986; McKinney et al, 1987; Hartley et al, 1988; Nishi and Miyake, 1989; Kaatsch et al, 1996; Dockerty et al, 1999; Schuz et al, 1999).

Recently, the results of a large case-control study conducted in the United States raised the possibility that conjugate vaccine against *Haemophilus influenzae* type b (Hib) could reduce the risk of childhood leukemia (Groves et al, 1999). Vaccination history was obtained from mothers or physicians of 439 case-control pairs, with cases diagnosed with childhood acute lymphoblastic leukaemia. An odds ratio of 0.55 (95% CI 0.35–0.87) was found for vaccination against Hib during the era when conjugate vaccine was predominant. No significant effect was detected for vaccine against Hib during the era when polysaccharide vaccination was in use, nor for any other vaccine. There was no a priori hypothesis, nor biological mechanism related to the Hib conjugate vaccine. It remains unclear if the finding was due to vaccination itself or avoidance of infection – alternative explanations also include chance, bias and confounding.

The aim of the present study was to evaluate the effect of different timing and number of doses of a conjugate Hib vaccine. A rare opportunity to compare different administration schemes within the setting of an intervention trial was provided by a nation-wide vaccination trial conducted in Finland in the 1980s (Eskola et al, 1990). This approach minimizes the effects of bias and confounding due to known and unknown risk factors.

*Received 14 February 2000*
*Revised 13 May 2000*
*Accepted 8 June 2000*

*Correspondence to:* A Auvinen

### MATERIAL AND METHODS

A nation-wide vaccine trial compared vaccination against *Haemophilus influenzae* type b (Hib) with multiple doses of a conjugate vaccine in infancy (early intervention) versus a single dose at the age of two years (late intervention) (Eskola et al, 1990). The vaccine consisted of heat-sized Hib capsular polysaccharide coupled to diphtheria toxoid (PRP-D) (Schneerson et al, 1980). All 114 000 children born in Finland between October 1, 1985 and August 31, 1987 were enrolled with participation rate of 98%. The children were allocated to the trial arms based on the date of birth. All children with an odd day of birth were assigned to the early intervention arm with vaccination at the ages 3, 4, 6 and a fourth dose given between 14 and 18 months, while children with an even day of birth were assigned to the late intervention arm and were not vaccinated until they reached the age of 24 months, when they received one dose. All the children also received standard vaccination regime including tuberculosis (BCG), diphtheria-pertussis-tetanus (DPT), inactivated polio (IPV) and measles-mumps-rubella (MMR) vaccine.

Information on the number of boys and girls born on odd and even dates during the periods of interest were obtained from the Finnish Population Registry (Table 1). Also, the number of

**Table 1** Numbers of children, person-years (PYRs) and leukaemia cases by period, odd versus even date and sex

| | Odd date | | Even date | |
|---|---|---|---|---|
| | **Boys** | **Girls** | **Boys** | **Girls** |
| No. | 32 689 | 31 227 | 31 390 | 29 845 |
| PYRs | 341 691 | 327 354 | 327 850 | 312 190 |
| Cases | 15 | 18 | 18 | 26 |

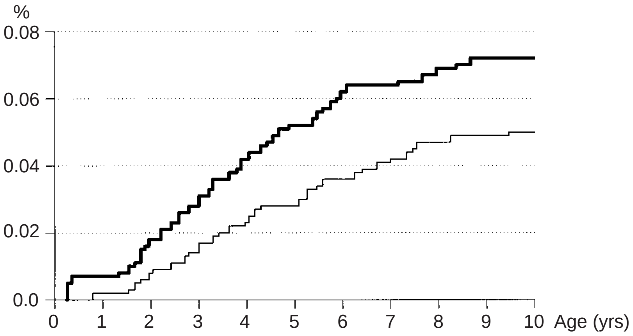

**Figure 1** Cumulative incidence of childhood leukaemia by age. Thick line: even date of birth, with a single vaccination at 24 months of age. Thin line: odd date of birth, with several immunizations between three and 18 months of age

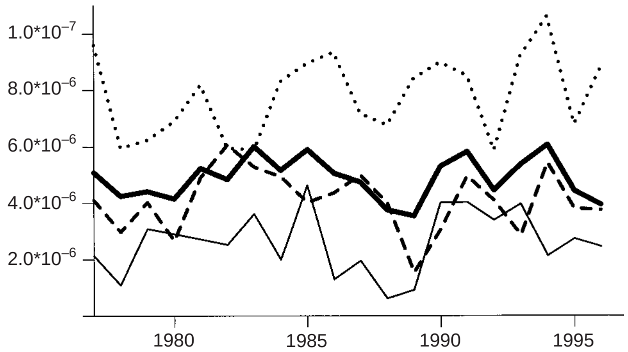

**Figure 2** Incidence rate of childhood leukaemia by age group in Finland in 1977–1996. Solid line: overall, 0–14 years, dotted line: 0–4 years, dashed line: 5–9 years, thin line: 10–14 years children alive in each group by year and sex was acquired. The length of follow-up (through 1996) was 9–11 years among children in the trial (born 1985–1987).

All cases of childhood leukaemia diagnosed in Finland were identified from the Finnish Cancer Registry, which is a nation-wide, population-based cancer registry (Teppo et al, 1994). Complete coverage of the cancer registry was ensured by crosschecking the case lists at all hospitals treating childhood leukaemia and from mortality records. All childhood leukaemia cases were histologically confirmed with bone marrow biopsy. In addition, incidence rates of childhood leukaemia in Finland in 1977–1996 by five-year age group were obtained from the Finnish Cancer Registry.

Poisson regression analysis of leukaemia incidence rates was conducted using the number of cases as the outcome variable and the natural logarithm of number of children at risk as an offset term (Breslow and Day, 1987). For comparison of the early and late vaccination groups, a binary variable based on odd or even date of birth was used. In the analysis of temporal trends, a continuous calendar year term was used.

### RESULTS

The numbers of children born during the trial period and person-years at risk are presented in Table 1.

A total of 77 leukaemia cases were diagnosed from birth through age 11 among subjects born during the trial period (1985–1987). Of them, 33 were observed among children born on an odd date, i.e., belonging to the early vaccination arm, while 44 were diagnosed among children in the late intervention arm. This corresponds to a relative risk of 0.72 (95% CI 0.46–1.13) for subjects born on an odd date. The cumulative incidence of childhood leukaemia in the two groups is shown in Figure 1.

Sixty-seven of the cases were acute lymphoblastic leukaemias (ALL). Among these cases 29 were born on odd days and 38 on even days. The corresponding relative risk for the early vaccination arm was 0.73 (95% CI 0.45–1.18).

When the analysis was restricted to cases diagnosed between the ages of three months and two years, i.e. the time period during which only the subjects in the early-intervention arm had been vaccinated, there were four leukaemia cases in the early vaccination arm and ten cases in the late vaccination arm. The corresponding relative risk was 0.47 (95% CI 0.09–2.59). For ALL alone, the numbers of cases in these groups were one and five (RR 0.19, 95% CI 0.00–1.72).

Incidence rate of childhood leukaemia remained practically constant, around 5 per 100 000 person-years in Finland in 1977–1996 (Figure 2). Poisson regression analysis showed no evidence for temporal trend (less than 1% change in relative risk per year), both overall (RR 1.00 per year, 95% CI 0.99–1.01) and for each five-year age groups (RR 1.01 per year, 95% CI 0.99–1.03 for 0–4 years of age, RR 1.00, 95% CI 0.98–1.02 for 5–9 years of age and RR 1.01, 0.98–1.03 for 10–14 years of age).

### DISCUSSION

There was a suggestion of a lower risk of childhood leukaemia among Finnish children who received multiple doses of *Haemophilus influenzae* type b conjugate vaccine in the first year of life than in those who received only a single dose at the age of two years. As expected, the effect was most pronounced prior to the age of two years, i.e. before the late intervention group was vaccinated. The design of the trial with allocation of intervention based on date of birth and prospective follow-up minimized the potential for bias and confounding. Very high participation rate reduced exposure misclassification to a minimum and complete ascertainment of cases was achieved by using the data from the Finnish Cancer Registry, based on clinical, pathological and cause of death records. However, we were not able to verify if the vaccination indeed took place for a given individual, even though it was very likely given the participation rate of 98%. We find it unlikely that selection bias could account for our results, because the analyses were conducted on the basis of intention to treat, i.e., non-participants were included in the study population.

The majority of previous studies have shown a larger proportion of unvaccinated children among leukaemia cases than controls (Kneale et al, 1986; McKinney et al, 1987; Hartley et al, 1988; Nishi and Miyake, 1989; Kaatsch et al, 1996; Schuz et al, 1999), but these findings have not been observed in all studies (Innis, 1965; Stewart and Hewitt, 1965; Salonen, 1976). Previously, only a German study has included the Hib vaccine among exposures studied, but the effects of Hib (or any other specific vaccination) were not reported separately (Kaatsch et al, 1996; Schuz et al, 1999). A US case-control study, however, found a statistically significant protective effect of Hib vaccination for the period when conjugate vaccine prevailed, but not for polysaccharide vaccine era (Groves et al, 1999).

No plausible biological mechanism has yet been identified, which could explain an apparent protective effect of early,

*© 2000 Cancer Research Campaign*

repeated Hib vaccination against childhood acute lymphoblastic leukaemia. It is possible that the vaccination stimulates the immune system in some way that reduces the subsequent risk of leukaemia, with the effect possibly depending on the age at first vaccination or the number of vaccinations. An infective basis of childhood leukaemia has been postulated and some limited evidence provided for it (Greaves, 1997; Kinlen, 1998; Smith et al, 1997). In particular, the delayed exposure hypothesis postulates that a small fraction of children are predisposed to leukaemia due to a somatic mutation, which occurs in utero. Early antigenic exposures are believed to protect against the development of leukaemia, while delayed exposure to the same antigens may actually trigger the preleukaemic clone to expand and progress to a leukaemia (Greaves, 1999). In addition, the incidence of childhood leukaemia has remained stable since the introduction of large-scale Hib vaccinations, even as the incidence of clinical and subclinical Hib infection has declined (Takala et al, 1991; Peltola et al, 1992; Takala et al, 1994). Also, invasive Hib infections are no more common than childhood ALL. Therefore, a protective effect due to prevention of Hib infection per se seems unlikely. However, vaccination decreases materially the prevalence of asymptomatic carriers (Takala et al, 1991).

Incidence rates of childhood leukaemia in Finland are comparable to other countries with nation-wide cancer registration (Parkin et al, 1998). No changes in incidence rates of childhood leukaemia have been observed in Finland following the introduction of a Hib vaccine programme in 1990. However, the type of vaccine used in the trial (PRP-D) was different from those used for the nation-wide vaccination policy (PRP-T in 1990–1991 and HbOC since 1991). The PRP-D and PRP-T vaccines are similar in that the polysaccharide from the capsule of Hib is covalently bound to a bacterial toxoid (a protein exotoxin denatured by heat treatment) from either *Clostridium tetani* (PRP-T) or *Corynebacterium diphtheriae* (PRP-D). In HbOC, the Hib oligosaccharide is coupled with the exotoxin of *Corynebacterium diphtheriae* strain CRM197; the exotoxin from this mutant strain does not require heat-treatment because it is nontoxic to humans. In view of the aforementioned differences between the PRP-D vaccine, which was used in the 1986–1987 trial, and the HbOC vaccine, which is currently in use, it may be informative to assess the incidence of childhood leukemia in Finland among subjects who participated in the nationwide clinical trial which compared early vaccination with PRP-D versus HbOC during 1988–1989 (Peltola et al, 1994).

Based on the results of studies published so far, we find it plausible that a protective effect could be attributable to bacterial polysaccharide antigens used in conjugate Hib vaccines. Most other pediatric vaccines including measles, mumps, rubella, polio, diphtheria, pertussis and tetanus consist of protein rather than polysaccharide antigens. Other bacterial polysaccharide vaccines such as pneumococcus or meningococcus vaccines are seldom administered to infants or young children.

In summary, our results based on a nation-wide vaccine trial suggest a protective effect of Hib conjugate vaccine against childhood leukaemia, when administered at an early age. It remains unclear, however, if the possible protective effect is due to Hib vaccination per se or avoidance of infection.

### ACKNOWLEDGEMENTS

We thank Dr Juhani Eskola from the National Public Health Institute, Helsinki, Finland for information on the vaccine trial and comments on the draft manuscript. We also thank Dr Martha Linet and Dr Robert Tarone, National Cancer Institute, Bethesda, Maryland for helpful comments and advice.

### REFERENCES

Breslow NE and Day NE (1987) *Statistical methods in cancer research*. Vol. II – The design and analysis of cohort studies, pp. 131–137. IARC Scientific Publications No. 82. International Agency for Research on Cancer: Lyon

Dockerty JD, Skegg DC, Elwood JM, Herbison GP, Becroft DM and Lewis ME (1999) Infections, vaccinations, and the risk of childhood leukaemia. *Br J Cancer* **80**: 1483–1489

Eskola J, Käyhty H, Takala AK, Peltola H, Rönnberg P-R, Kela E, Pekkanen E, McVerry PH and Mäkelä PH (1990) A randomized, prospective field trial of a conjugate vaccine in the protection of infants and young children against invasive *Haemophilus influenzae* type b disease. *N Engl J Med* **323**: 1381–1387

Greaves MF (1997) Aetiology of acute leukaemia. *Lancet* **349**: 344–349

Greaves M (1999) Molecular genetics, natural history and the demise of childhood leukemia. *Eur J Cancer* **35**: 173–185

Groves FD, Gridley G, Wacholder S, Shu X-O, Robison LL, Neglia J and Linet MS (1999) Infant vaccinations and risk of childhood acute lymphoblastic leukaemia in the United States. *Br J Cancer* **81**: 175–178

Hartley AL, Birch JM, McKinney PA, Blair V, Teare MD, Carrette J, Mann JR, Stiller CA, Draper GJ, Johnston HE, Cartwright RA and Waterhouse JAH (1988) The Inter-Regional Epidemiological Study of Childhood Cancer (IRESCC): past medical history in children with cancer. *J Epidemiol Community Health* **42**: 235–242

Innis MD (1965) Immunisation and childhood leukaemia. *Lancet* **1**: 605

Kaatsch P, Kaletsch U, Krummenauer F, Meinert R, Miesner A, Haaf G and Michaelis J (1996) Case-control study on childhood leukaemia in Lower Saxony, Germany. *Klin Pädiatr* **208**: 179–185

Kinlen LJ (1998) Infection and childhood leukaemia. *Cancer Causes Control* **9**: 237–239

Kneale GW, Stewart AM and Wilson LMK (1986) Immunizations against infectious diseases and childhood cancer. *Cancer Immunol Immunother* **21**: 129–132

McKinney PA, Cartwright RA, Saiu JMT, Stiller CA, Draper GJ, Hartley AL, Hopton PA, Birch JM and Waterhouse CA (1987) The Inter-Regional Epidemiological Study of Childhood Cancer (IRESCC): a case-control study of aetiological factors in leukaemia and lymphoma. *Arch Dis Child* **62**: 279–287

Nishi M and Miyake H (1989) A case-control study of non-T cell acute lymphoblastic leukaemia of children in Hokkaido, Japan. *J Epidemiol Comm Health* **43**: 352–355

Parkin DM, Kramarova E, Draper GJ, Masuyer E, Michaelis J, Neglia J, Qureshi S and Stiller CA (1998) *International Incidence of Childhood Cancer, Vol. II*. IARC Scientific Publication No. 144. International Agency for Research on Cancer: Lyon, France.

Peltola H, Kilpi H and Anttila N (1992) Rapid disappearance of *Haemophilus influenzae* type b meningitis after routine childhood immunisation with conjugate vaccines. *Lancet* **340**: 592–594

Peltola H, Eskola J, Käyhty H, Takala AK and Mäkelä PH (1994) Clinical comparison of the *Haemophilus influenzae* type b polysaccharide-diphtheria toxoid and the oligosaccharide-CRM197 protein vaccines in infancy. *Arch Pediatr Adolesc Med* **148**: 620–625

Salonen T (1976) Prenatal and perinatal factors in childhood cancer. *Ann Clin Res* **7**: 27–42

Schneerson R, Barrera O, Sutton A and Robbins JB (1980) Preparation, characterization, and immunogenicity of *Haemophilus influenzae* type b polysaccharide-protein conjugates. *J Exp Med* **152**: 361–376

Schuz J, Kaletsch U, Meinert R, Kaatsch P and Michaelis J (1999) Association of childhood leukaemia with factors related to the immune system. *Br J Cancer* **80**: 585–590

Smith MA, Chen T and Simon R (1997) Age-specific incidence of acute lymphoblastic leukaemia in U.S. children: In utero initiation model. *J Natl Cancer Inst* **89**: 1542–1544

Stewart AM and Hewitt D (1965) Aetiology of childhood leukaemia. *Lancet* **2**: 789–790

Takala AK, Eskola J, Leinonen M, Käyhty H, Nissinen A, Pekkanen E and Mäkelä PH (1991) Reduction of oropharyngeal carriage of *Haemophilus influenzae* type b in children immunized with an Hib conjugate vaccine. *J Infect Dis* **164**: 982–986

Takala AK, Peltola H and Eskola J (1994) Disappearance of epiglottitis during large-scale vaccination with *Haemophilus influenzae* type b conjugate vaccine among children in Finland. *Laryngoscope* **104**: 731–735

Teppo L, Lehtonen M and Pukkala E (1994) Data quality and quality control of a population-based cancer registry. *Acta Oncol* **33**: 365–369

 © 2000 Cancer Research Campaign
